# Identifying Factors Associated With HIV Viral Suppression and Health Care Outcomes in the Florida Cohort Study Wave 3: Protocol for a Prospective Cohort Study

**DOI:** 10.2196/69702

**Published:** 2025-09-25

**Authors:** Rebecca J Fisk-Hoffman, Christina E Parisi, Nanyangwe Siuluta, Preeti Manavalan, Lori A Bilello, Colby Cohen, Jessy Devieux, Gladys Ibanez, Jennifer Kuretski, Charurut Somboonwit, Maya Widmeyer, Zhi Zhou, Robert L Cook, Florida Cohort Wave 3 Team

**Affiliations:** 1 Department of Epidemiology College of Public Health and Health Professions & College of Medicine University of Florida Gainesville, FL United States; 2 Department of Psychiatry Perelman School of Medicine University of Pennsylvania Philadelphia United States; 3 College of Medicine University of Florida Gainesville United States; 4 College of Medicine - Jacksonville University of Florida Health Science Center Jacksonville United States; 5 HIV/AIDS Section Bureau of Communicable Diseases Florida Department of Health Tallahassee United States; 6 Department of Health Promotion and Disease Prevention Robert Stempel College of Public Health and Social Work Florida International University Miami United States; 7 Robert Stempel College of Public Health and Social Work Florida International University Miami United States; 8 Midway Specialty Care Center West Palm Beach, FL United States; 9 Department of Internal Medicine College of Medicine University of South Florida Tampa United States; 10 Comprehensive Health Care Melbourne, FL United States; 11 see Acknowledgments

**Keywords:** HIV, sustained virologic response, cohort studies, alcohol consumption, epidemiologic research design

## Abstract

**Background:**

Ending the HIV epidemic remains a high public health priority, and the state of Florida continues to have high HIV prevalence and incidence.

**Objective:**

This protocol aims to identify factors associated with the HIV care continuum and HIV-related comorbidities, with a focus on the impacts of alcohol use.

**Methods:**

The Florida Cohort study wave 3 enrolled people with HIV aged 18 years or older from 9 clinical, case management, and community settings across Florida from 2020 to 2023. All participants completed a baseline questionnaire, and most (769/836, 92%) completed additional questionnaires at baseline and/or approximately 1 year after baseline. Data on HIV care and treatment, mental health, substance use, stigma, and technology were collected in the baseline questionnaire. Additional questionnaires covered alcohol use, gender identity, pet ownership, stigma and discrimination, antiretroviral therapy preferences, and the impacts of the COVID-19 pandemic. Questionnaire data were securely linked to HIV care continuum variables from Florida’s state HIV monitoring system.

**Results:**

Overall, the study enrolled 836 people with HIV. Among them, 397 (47.5%) were non-Hispanic Black, 131 (15.7%) were Hispanic, 505 (60.4%) were assigned male sex at birth, and 487 (58.3%) were aged above 50 years. Most (n=769, 92%) participants were linked to the state HIV reporting system and will be followed for up to 5 years to monitor HIV outcomes. A total of 31 (94% of 33 eligible) participants completed the gender identity questionnaire, 230 (91.3% of 252 eligible) completed the alcohol questionnaire, 287 (91.7% of 313 eligible) completed the COVID-19 questionnaire, 221 (85% of 260 eligible) completed the pet questionnaire, 461 (87.6% of 526 eligible) completed the stigma and discrimination questionnaire, and 210 (85.7% of 245 eligible) completed the antiretroviral therapy preference questionnaire.

**Conclusions:**

This study provides opportunities to monitor changes in HIV-related outcomes as well as relevant attitudes, behaviors, and health care preferences; however, it has some limitations in terms of representativeness and tracking longitudinal outcomes.

**International Registered Report Identifier (IRRID):**

DERR1-10.2196/69702

## Introduction

### Background

In 2019, there were 13.2 HIV diagnoses per 100,000 people in the United States. The Ending the HIV Epidemic (EHE) initiative was announced with the goal of reducing new HIV infections by 90% by 2030 [1]. A key pillar of the EHE is helping more people with HIV achieve and sustain viral suppression to decrease transmission of HIV [2]. Florida, an epicenter of the HIV epidemic in the United States, has high rates of HIV incidence (19.9 per 100,000 people) and prevalence (578.7 per 100,000 people) in both urban and rural settings and in people with a diversity of racial and cultural backgrounds [3-5]. When the Florida Cohort study wave 3 began in 2019, only 70% of the 123,087 people with HIV in Florida had achieved viral suppression [5]. Identifying barriers that prevent people with HIV from sustaining viral suppression and achieving other care continuum steps, as well as facilitators associated with successful HIV outcomes, within Florida is needed to achieve the ambitious targets set by the EHE.

Numerous factors at the individual, interpersonal, community, and policy levels influence HIV viral suppression [6-8], including alcohol use, other drug use, housing status, mental health conditions, HIV stigma, having low income, and the lack of insurance coverage, among others [9-13]. Trends in alcohol use are changing as alcohol use among women has increased, and the COVID-19 pandemic has increased alcohol use in the general population [14,15]. At the same time, the influence of alcohol use on viral suppression may be lessening as antiretroviral therapy (ART) regimens become simpler and more forgiving [10,16,17]. However, alcohol use and ART can both negatively impact liver function. Therefore, monitoring alcohol use and interest in alcohol-related interventions remains important for improving the health of people with HIV [18].

Many of these factors that directly or indirectly impact viral suppression are infrequently recorded in medical records or surveillance data and can only be assessed by directly administering questionnaires to people with HIV. While there are many ongoing cohort studies of HIV, the Florida Cohort study is the only one focused on monitoring HIV-related factors across an entire state. In the previous iteration of the Florida Cohort study (wave 2), we collected data from more than 900 people with HIV between 2014 and 2018, linking data from individual questionnaires, medical records, and statewide HIV surveillance [19]. From these data, our team identified several factors associated with poor HIV viral suppression, including heavy alcohol consumption, other substance use, depression and anxiety symptoms, and suboptimal ART adherence [20-25]. We also examined several dimensions of HIV stigma and found that only health care–associated experiences of stigma and discrimination were significantly associated with poor viral suppression in this sample [26]. Many of these factors, attitudes, and behaviors are not well captured in medical records and may change over time. Moreover, changes in health care policies, HIV medications, alcohol and other substance use, and temporal events (such as pandemics) may correlate with changes in individual behaviors, health conditions, or attitudes and beliefs over time. Therefore, ongoing monitoring is needed to assess their continued impact on HIV care outcomes.

### This Study

The Florida Cohort study wave 3 focused on factors that are not readily assessed via medical records, including alcohol and other substance use, experiences of stigma and discrimination, and mental health symptoms [27,28]. We sought to capture additional measures related to alcohol consumption and potential interventions that address alcohol use disorder. On the basis of feedback from community members and interests of research investigators, we also included new questions related to HIV stigma and discrimination, barriers to substance use and mental health services, interest in emerging options for health care such as telehealth, and challenges related to ART adherence. We also received supplements to study the impacts of the COVID-19 pandemic and HIV care among gender-diverse people with HIV; therefore, additional questionnaire items were added to capture these issues. Items were also added to support topics related to graduate student research. Thus, the overall goals of the Florida Cohort study wave 3 were to identify barriers to and facilitators of HIV viral suppression and other steps of the HIV care continuum, focusing on alcohol use, and monitor changing attitudes and beliefs that influence HIV care outcomes. This paper describes recruitment, study measures, and baseline characteristics of the study population in accordance with the CHERRIES (Checklist for Reporting Results of Internet E-Surveys) and STROBE (Strengthening the Reporting of Observational Studies in Epidemiology) checklists (see Multimedia Appendices 1 and 2).

## Methods

### Study Design and Study Population

The Florida Cohort study is a prospective cohort study that obtained baseline questionnaire data from study participants and linked questionnaire data to statewide HIV surveillance data, including HIV viral load, which will be tracked for at least 5 years after enrollment. The baseline questionnaire included several core measures. Participants had the option to complete additional questionnaires at baseline and follow-up questionnaires at 12 months based on the timing of enrollment and staff resources available at the different recruitment sites. Participants were eligible if they were aged 18 years or above, were living with HIV, and received most of their HIV-related care in Florida. All recruitment settings provided the study in English, whereas some sites offered the study in Spanish, and 1 location offered the study in Haitian Creole. We sought to enroll a sample of people with HIV who are similar to the overall population of people with HIV in Florida in terms of age, gender, race, and ethnicity, including Haitian Creole and Spanish-speaking individuals [5]. The enrollment target was 1000 people with HIV, which included at least 200 (20%) people who met the criteria for at-risk drinking (assessed via the Alcohol Use Disorders Identification Test–Consumption), 50 (5%) people who spoke Haitian Creole, and 150 (15%) people living and receiving care in rural areas [29]. These populations were underrepresented in the previous versions of the Florida Cohort study but are key populations for addressing the HIV epidemic in Florida.

### Recruitment Settings

**Figure 1:**
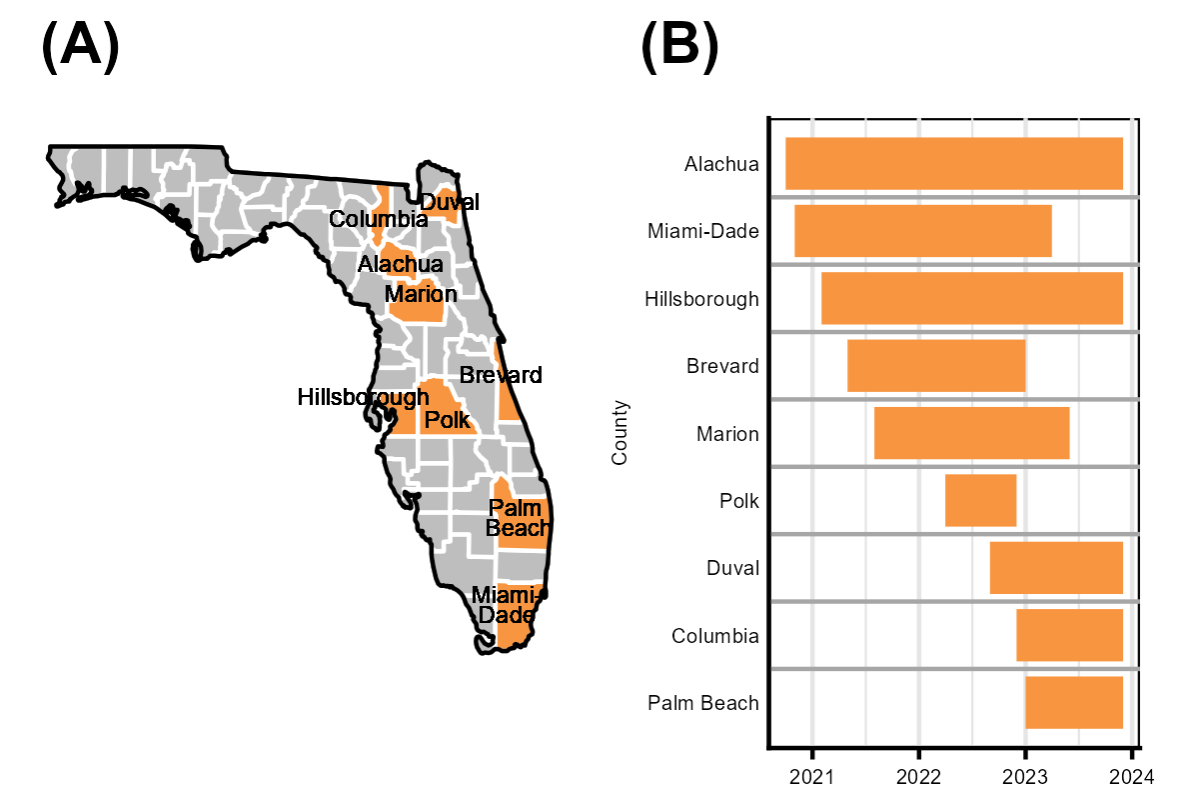
(A) Locations and (B) timeline of recruitment for the Florida Cohort Wave 3 Study.

Recruitment began in October 2020 and continued through December 2023 and used a convenience sampling method. The study recruited participants from 9 counties in Florida (major cities noted in parentheses): Alachua (Gainesville), Columbia, Marion (Ocala), Duval (Jacksonville), Hillsborough (Tampa), Brevard (Melbourne), Palm Beach, Polk, and Miami-Dade (Miami; Figure 1).

Most (828/836, 99%) participants were recruited from clinical care or community settings, including public health, academic, and community-based HIV clinics, and case management agencies. In Alachua, Columbia, Marion, Hillsborough, and Polk counties (Figure 1), recruitment occurred primarily within the Florida Department of Health (FDOH) clinics, where potential participants either provided a phone number to be contacted by a research team member or met with a study staff member who was present at the clinic. Recruitment sites in north-central Florida also included an academic infectious disease clinic in Alachua County and mailing flyers to Ryan White HIV/AIDS Program clients in area 3/13. In Duval County, recruitment focused on an HIV case management agency. In Miami-Dade, Brevard, and Palm Beach counties, recruitment focused on community-based Ryan White HIV/AIDS Program–funded clinics. Recruitment strategies in Miami-Dade also included displaying advertisements on buses. Spanish versions of the study were available in Polk and Miami-Dade counties, and the Creole version was offered in Palm Beach County. Initial enrollment procedures were modified to support remote enrollment after the start of the COVID-19 pandemic in 2020, including procedures to confirm eligibility, obtain consent, and enroll people via telephone or videoconferencing. By the end of 2020, most recruitment sites were able to enroll participants in person.

Additional study funding was obtained to provide a more representative sample of gender-diverse participants. To increase enrollment of gender-diverse participants, we provided recruitment information to medical and support agencies supporting transgender and gender nonconforming individuals in Alachua and Miami-Dade counties and worked with a private recruitment resource (TrialFacts) to publish recruitment advertisements on social media in the fall of 2023. Individuals who visited Facebook and Instagram within Florida were shown an advertisement for the study and could voluntarily click on the advertisement to be redirected to a prescreening form managed by TrialFacts. Those who indicated that they were living with HIV shared their contact information with the research team for routine screening and consent procedures, if eligible. Ancillary study protocols enrolled gender-diverse people without HIV, and details of these procedures will be described elsewhere.

### Screening

All participants completed a brief screening form to confirm that they met the age and HIV status criteria and estimate the number of surveys available to them as a part of the study. These screenings could be performed in person or over the phone. Once eligibility was confirmed, they were scheduled for an appointment to obtain consent. The consent form was accessible through a unique link that was emailed to them, or they could review and sign a paper copy of the consent form. Paper consent forms could be mailed and reviewed over the phone or could be provided in person if the potential participant and recruiter were meeting face-to-face. Those who were emailed the consent form could proceed to the questionnaires through that same link after providing consent.

### Questionnaire Development

The study team reviewed the questionnaires developed for the Florida Cohort study wave 2 and identified areas that were underexplored or not explored in previous iterations of the study [19]. Members of the research team then reviewed the relevant literature on the areas identified for further exploration in wave 3 to determine measures to assess these domains. An updated version of the questionnaire was then circulated to the wider research team to identify other areas of exploration, and researchers could suggest measures for inclusion. The initial version of the questionnaire was then reviewed by community consultants and revised accordingly. The surveys were then built in Qualtrics (Qualtrics International Inc) and tested by the research team to verify that the wording and skip logic were correct. Subsequently, the questionnaire was piloted among people with HIV enrolled in another Southern HIV and Alcohol Research Consortium (SHARC) study and revised based on participant feedback. Data from the pilot study were not included in the final dataset. Subsequent questionnaires were reviewed by the research team and community consultants before being added to the study.

### Data Collection

Participants were encouraged to complete the study questionnaires online using a Qualtrics-based survey that could be completed either at the recruitment location or at home. The Qualtrics survey showed 1 or 2 questions per page, and the number of pages varied based on participants’ responses. They were allowed to skip questions after dismissing a prompt to go back and answer any blank questions on the page. Participants could also return to the survey and change previous responses. Cookies were enabled so that participants could return to the same page that they were last on if they used the same device and browser.

Participants also had the option to complete questionnaires on paper or have the research assistant read the questions and enter the answers in Qualtrics. The Haitian Creole version of the survey was administered orally by a research assistant who was fluent in Haitian Creole. The order of questions was the same regardless of the administration method.

### Questionnaires

#### Core Measures

All participants at all study sites completed a baseline questionnaire that included items on sociodemographic characteristics, alcohol and drug use, mental health symptoms, HIV care and treatment, and potential use of technology for health (Textbox 1). In March 2022, the baseline questionnaire was updated to include additional questions about interest in and barriers to mental health interventions, psychosocial distress, long-acting injectable ART, characteristics of sexual partners, reproductive history and health, and the use of mobile apps. These same items were included in the 12-month questionnaire for people who enrolled before this change. The baseline questionnaire at sites that only offered a single baseline questionnaire included some additional items related to alcohol consumption and HIV care that were included in separate questionnaires at most settings.

**Summary of measures included in the Florida Cohort study questionnaires. The original source of questionnaire items is included unless they are items created specifically for this study.**

**Baseline questionnaire**

- Demographics
- Socioeconomic status
- Symptoms of depression and anxiety [30,31]
- Receipt of and interest in mental health care
- Distress [32]
- Antiretroviral therapy (ART) and HIV care
- Interest in long-acting ART
- Recent alcohol use [29]
- Heaviest alcohol use [33]
- Other substance use
- Medical marijuana
- Sexual activity
- Women’s Health (pregnancy, contraception, and screening)
- Use of computers and mobile devices (telehealth use, internet access, and app use)
- COVID-19 (testing and vaccination history) [34,35]

**Alcohol questionnaire**

- Recent alcohol use [36] (this topic was also included in the baseline questionnaire for participants at study sites where only a single baseline questionnaire was offered)
- Motivations for drinking [37]
- Negative consequences of alcohol use [38]
- Attempts to quit
- Drinking for pain management
- Withdrawal symptoms [39]
- Interest in alcohol treatment and intervention options

**Gender identity questionnaire**

- Gender identity and expression [40]
- Discrimination as a barrier to HIV care
- Gender-based discrimination [41]
- Access to gender-affirming care [41]
- Receipt of gender-affirming care [41]

**Extended questionnaire**

- Employment
- Safety net program use
- Evictions
- Other mental health conditions [42]
- Negative urgency [43]
- Gambling
- Care satisfaction
- ART access
- Other medical care and hospitalizations
- Disclosure of HIV status
- Lifetime alcohol use [33]
- History of alcohol treatment and barriers to care (this topic was also included in the baseline questionnaire for participants at study sites where only a single baseline questionnaire was offered)
- Sexually transmitted infection diagnosis (this topic was also included in the baseline questionnaire for participants at study sites where only a single baseline questionnaire was offered)
- New sex partners
- Adverse life events [44]
- Interest in alternative therapies
- Past incarceration and HIV diagnosis while incarcerated

**COVID-19 questionnaire**

- Impacts of the COVID-19 pandemic
- COVID-19 testing
- Infection history
- Vaccination history and beliefs [34,35]
- Loneliness [45]

**Pet questionnaire**

- Pet ownership
- Pet services
- Impacts on health and health care [46]
- Social support [47]

**Stigma questionnaire**

- HIV-related stigma [48]
- Enacted stigma
- Disclosure of HIV status (this topic was also included in the baseline questionnaire for participants at study sites where only a single baseline questionnaire was offered)
- Discrimination in health care
- Experiences with disease investigation specialists

**ART preference questionnaire**

- Preference for long-acting HIV treatment options

**12-month follow-up questionnaire**

- Socioeconomic status
- Symptoms of depression and anxiety [30,31]
- Receipt of and interest in mental health care
- ART and HIV care
- Other medical care and hospitalizations
- Recent alcohol use [29]
- Other substance use
- Sexually transmitted infection diagnoses
- New partners
- Women’s health (pregnancy, contraception, and screening)
- Telehealth use and access
- Payment app use
- COVID-19 positivity and vaccination history

#### Additional Questionnaires

**Figure 2:**
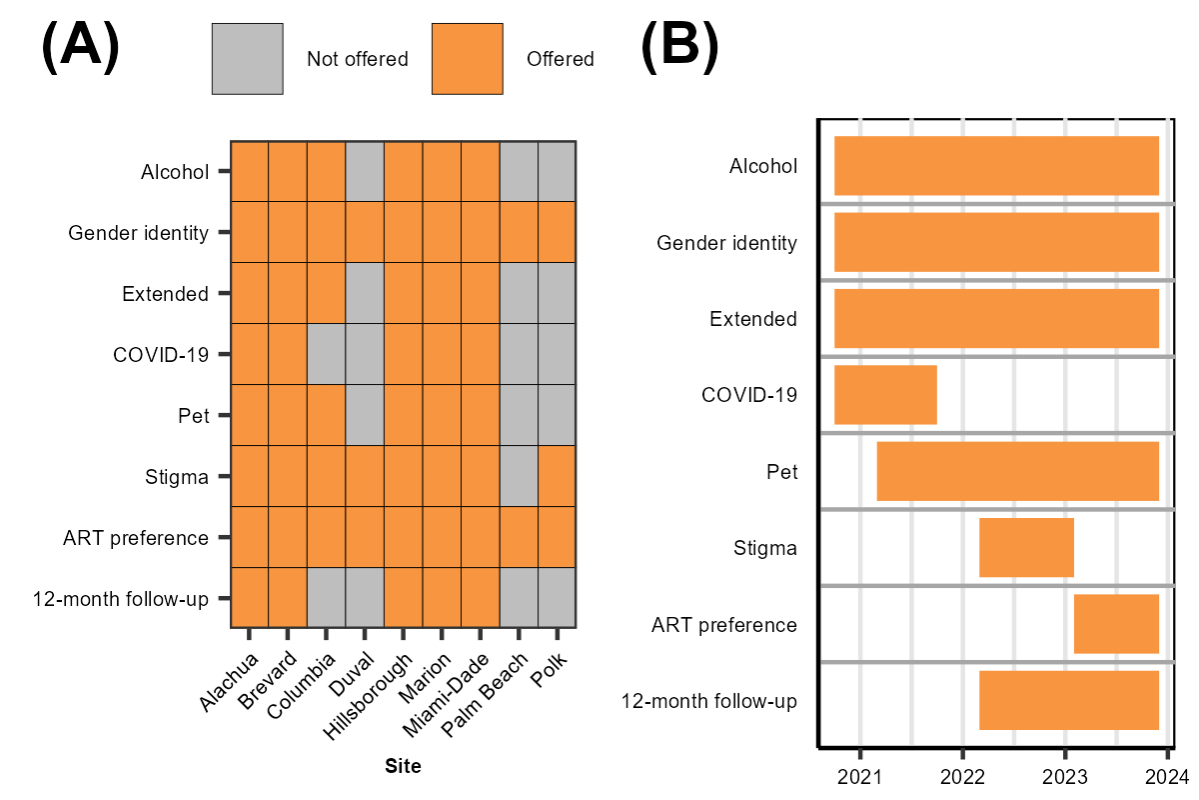
Questionnaire availability by (A) geographic location and (B) time period for the Florida Cohort Wave 3 Study.

After completing the baseline questionnaire, participants were invited to complete 1 or more additional questionnaires at baseline (Textbox 1 and Figure 2). The availability of additional questionnaires depended on the study site and the time at which participants enrolled in the study (Figure 2).

An overview of each additional questionnaire is provided in Textbox 2, and Textbox 1 provides more information on questionnaire content.

All questionnaires were available in English or Spanish, except for the ART preference questionnaire, which was only available in English. The core and 12-month follow-up surveys were also available in Haitian Creole.

**Overview of the additional questionnaires.**

- The alcohol questionnaire focused on recent alcohol use and treatment interest. Measures included the 10-item Alcohol Use Disorder Identification Test (assesses drinking quantity and frequency and symptoms of alcohol dependence in the preceding 12 months), the revised short form of the Drinking Motives Questionnaire, a shortened version of the Sexual Motivations for Drinking Questionnaire, the Short Inventory of Problems–Revised, and the revised Clinical Institute Withdrawal Assessment of Alcohol Scale [36-39]. This questionnaire was available to participants completing the baseline visit who reported drinking alcohol at least twice a month in the preceding 12 months.
- The gender identity questionnaire focused on gender identity and related care. This questionnaire was available to participants completing the baseline visit who identified as a gender different from the one that aligned with their sex assigned at birth.
- The extended questionnaire included additional questions from researchers collecting preliminary data for other grants and questions from trainees to support dissertation research. This questionnaire was available to participants completing the baseline visit.
- The COVID-19 questionnaire focused on the impacts of the COVID-19 pandemic and practices. This questionnaire was available to participants completing the baseline visit.
- The pet questionnaire focused on pet ownership and its impacts on quality of life and medical care. This questionnaire was available to participants completing the baseline visit who reported having at least 1 pet.
- The stigma questionnaire focused on HIV-related stigma and discrimination in health care. This questionnaire was available to participants completing the baseline or 12-month follow-up visit.
- The antiretroviral therapy preference questionnaire focused on identifying antiretroviral therapy characteristics of greatest importance to people with HIV using a discrete choice experiment. This questionnaire was available to participants completing the baseline or 12-month follow-up visit.
- The 12-month follow-up questionnaire repeated measures of key variables from the baseline questionnaire. This questionnaire was available to participants who completed a baseline visit before February 2023.

### Key Variables

Alcohol use in the preceding 12 months was assessed using the Alcohol Use Disorders Identification Test–Consumption, a 3-item measure that includes drinking quantity and frequency and heavy drinking episodes. At-risk drinking was defined as a score of 3 or greater for women and 4 or greater for men. Low-risk drinking was defined as any alcohol use below these cutoff scores [29].

Rurality was determined by linking rural-urban commuting area codes to participants’ zip code data [49]. We grouped the codes into 2 categories, so that all rural-related codes were grouped together [50].

### Training and Quality Assurance

All study recruiters received training before the enrollment of participants. In addition to training required by the institutional review boards (IRBs), training included sessions on the enrollment process; a review of the questionnaires; an overview of the use of REDCap (Research Electronic Data Capture; Vanderbilt University) for data collection and storage; secure communication using Microsoft Teams; and a discussion of ethical issues relevant to the study, including the informed consent process. Before actual enrollment, each recruiter was observed conducting a mock enrollment, and additional training was provided to certain staff who might conduct interviewer-administered versions of the questionnaire involving reading the questions out loud to participants.

### State Surveillance Data and Medical Records

After obtaining participant consent, we securely linked survey participants to statewide FDOH HIV surveillance data under a formal data use agreement with FDOH. The linkage procedures have been previously described in detail [19]. The surveillance data include dates and results from laboratory tests for CD4 and HIV viral load tests from the Enhanced HIV/AIDS Reporting System (eHARS) managed by FDOH. Data are also linked to the National Death Index, which allows the team to examine survival over time. These results can be used to calculate aspects of the HIV care continuum, including engagement in care and HIV viral suppression. For the subset of participants who were recruited from FDOH clinics, the team also received data from electronic medical records managed by the FDOH, including information on medication prescriptions and medical condition diagnoses.

### Follow-Up

The survey follow-up ended with administration of the 12-month follow-up questionnaire for participants at the sites where survey follow-ups were offered. Study staff attempted to contact participants who were enrolled at a site where the 12-month follow-up questionnaire was offered at 6 months to update their contact information as needed. For the 12-month follow-up questionnaire, up to 6 contact attempts were made using at least 2 forms of contact given at baseline before the participant was considered lost to follow-up.

All Florida Cohort study wave 3 participants will be followed in eHARS and through FDOH medical records through 2028, allowing for longitudinal data collection for these HIV-related outcomes. FDOH and eHARS records will be updated yearly.

### Data Management and Data Sharing

All study data are maintained on secure servers at the University of Florida (UF). All enrollment, consent, and visit log data were entered into REDCap. Consent forms completed on paper were scanned into REDCap. Deidentified paper questionnaires were entered into Qualtrics by research study staff using a double data entry procedure to minimize data entry errors. Participant data were excluded from the final dataset if participants had previously completed the same study questionnaire, when questionnaires had substantial missing information, or when data entry seemed highly infeasible (eg, completing an entire questionnaire in less than 5 minutes when most other participants took 20 or more minutes to complete it; 8 participants). Data managers also reviewed the data to identify likely duplicate records. This included participants who gave different names but the same phone number or who had the same first and last name but with varying middle names or initials. IP addresses were not collected and could not be used to identify duplicates.

Deidentified datasets that do not contain FDOH information are available to investigators who submit a research plan through the SHARC Concepts System [51]. This requires researchers to submit the study rationale, aims and hypotheses, and a detailed research plan, which are reviewed by a team of researchers involved with SHARC and the Florida Cohort study wave 3. The reviewers provide feedback and can request changes and clarifications before approving the research plan and sharing the data. Data that include information from FDOH surveillance data or medical records will require separate data use agreements and approvals from FDOH.

### Planned Analyses

The Florida Cohort study wave 3 allows for both longitudinal and cross-sectional analyses. Cross-sectional analyses include estimating the prevalence of at-risk alcohol use, identifying common motivations for alcohol use and negative outcomes related to alcohol use, and assessing interest in different alcohol-related interventions and treatments. We will also estimate the prevalence of other substance use, unstable housing, symptoms of anxiety and depression, and interactions between these conditions and alcohol use. For the longitudinal analyses, we will assess how alcohol use influences engagement in HIV care and viral suppression over time and how the COVID-19 pandemic impacted alcohol use among people with HIV. Additional analyses will be carried out to meet the goals of the junior faculty and student projects. Examples of these analyses include assessing the impact of psychosocial distress on ART adherence, assessing interest in mobile phone–based mental health interventions, and assessing the impacts of interpersonal violence on HIV care engagement.

### Ethical Considerations

The IRB at the UF served as the IRB of record for this multisite study and approved all study activities before implementation (201801680). The IRBs at the FDOH, Florida International University, and the University of South Florida ceded to the UF. All participants provided written informed consent before participation, and individuals had the opportunity to opt out of the study. Identifiable participant data were maintained separately from research data on secure servers at the UF, and limited datasets were prepared by the data management team to ensure participant privacy and confidentiality. Data from the FDOH, including eHARS data, were only accessible through ResVault. Participants received incentive payments via gift cards or cash, depending on the study setting. The amount of compensation varied based on the number of questionnaires completed and the length of those questionnaires (ranging from US $10 to US $25 per questionnaire). Total compensation ranged between US $25 and US $120.

## Results

Between October of 2020 and December of 2023, a total of 1291 people were screened. Of these, 172 (13.3%) were found to be ineligible, 34 (2.6%) were not interested, 179 (13.9%) provided consent but never completed the baseline questionnaire, 26 (2%) were withdrawn by the research team for various reasons (eg, duplicates or suspicious timing), and 44 (3.4%) were enrolled in a parallel study. In total, 836 (64.8%) people with HIV enrolled in the Florida Cohort study wave 3 and completed a baseline questionnaire. Most participants with HIV identified as non-Hispanic Black (n=397, 47.5%) and male (n=505, 60.4%) and were aged 50 years or older (n=487, 58.3%), and 131 (15.7%) identified as Hispanic. Most people with HIV reported some alcohol use, with 238 (28.5%) participants reporting at-risk use and 308 (36.8%) reporting low-risk use (Table 1).

Of the 836 participants with HIV, most (n=759, 90.8%) completed the modules in English, 27 (3.2%) participants completed the modules in Spanish, and 50 (6%) completed the modules in Haitian Creole.

Completion rates for additional questionnaires ranged from 94 to 85% (see Table 2). The follow-up rate for those eligible for the 12-month follow-up questionnaire was 54.7% (320/585). In addition, 97.5% 815/836) of the sample was linked to eHARS, and 63.3% (529/836) were linked to FDOH’s electronic health record system.

**Table 1:** Characteristics of people with HIV enrolled in the Florida Cohort study wave 3 (2020-2023) and of all people with HIV in Florida in 2022 [5].

Characteristic			People with HIV enrolled in this study (N=836), n (%)		People with HIV in Florida in 2022, %
**Year of enrollment**					
	2020	38 (4.5)		—^a^	
	2021	324 (38.8)		—	
	2022	296 (35.4)		—	
	2023	178 (21.3)		—	
**Age (y)**					
	13-19	0 (0)		0.3	
	20-24	20 (2.4)		1.8	
	25-29	51 (6.1)		5	
	30-34	72 (8.6)		8.6	
	35-39	66 (7.9)		8.9	
	40-44	80 (9.6)		9.2	
	45-49	60 (7.2)		9.4	
	50-54	119 (14.2)		12	
	55-59	146 (17.5)		15	
	>60	222 (26.6)		29	
**Sex assigned at birth**					
	Male	505 (60.4)		74	
	Female	331 (39.6)		26	
**Gender (self-identified)**					
	Cisgender man	473 (56.6)		74	
	Cisgender woman	328 (39.2)		26	
	Transgender man	2 (0.2)		0	
	Transgender woman	20 (2.4)		0.5	
	Nonbinary	12 (1.4)		0	
**Race and ethnicity**					
	American Indian or Alaska Native	6 (0.7)		0.1	
	Asian	2 (0.2)		0.7	
	Hispanic	131 (15.7)		27.1	
	Native Hawaiian or Pacific Islander	0 (0)		0.1	
	Non-Hispanic Black	397 (47.5)		43.2	
	Non-Hispanic White	255 (30.5)		27.5	
	Multiracial or other	40 (4.8)		1.3	
**Rurality**					
	Rural, small town, or large rural town	52 (6.2)		—	
	Urban	779 (93.2)		—	
**Language of survey completion**					
	English	759 (90.8)		—	
	Spanish	27 (3.2)		—	
	Haitian Creole	50 (6)		—	
**Education**					
	Less than high school	204 (24.4)		—	
	High school or general educational development	245 (29.3)		—	
	At least some college	386 (46.2)		—	
**Alcohol use**					
	At risk^b^	238 (28.5)		—	
	Not at risk	308 (36.8)		—	
	None	284 (34)		—	
**Site**					
	Alachua, Columbia, and Marion	251 (30)		—	
	Hillsborough	155 (18.5)		—	
	Miami-Dade	80 (9.6)		—	
	Polk	74 (8.9)		—	
	Palm Beach	83 (9.9)		—	
	Brevard	128 (15.3)		—	
	Duval	57 (6.8)		—	
	Online	8 (1.1)		—	

^a^Not available.

^b^At-risk drinking was defined as scoring 3 or greater on the Alcohol Use Disorder Identification Test–Consumption measure for people with HIV assigned female at birth and 4 or greater for people with HIV assigned male at birth.

**Table 2:** Completion of the additional Florida Cohort study questionnaires.

Questionnaire	Eligible participants, n	Completed questionnaires, n (%)
Extended	590	545 (92.4)
Gender identity	33	31 (94)
Alcohol	252	230 (91.3)
COVID-19	313	287 (91.7)
Pet	260	221 (85)
Stigma	526	461 (87.6)
Antiretroviral therapy preference	245	210 (85.7)
12-month follow-up	585	320 (54.7)

## Discussion

### Anticipated Findings

The overall goals of the Florida Cohort study wave 3 were to identify barriers to and facilitators of HIV viral suppression and other steps of the HIV care continuum and monitor changing attitudes and beliefs that influence HIV care outcomes and beliefs among a diverse group of people with HIV in Florida. We also sought to learn more about alcohol consumption patterns and potential treatment interventions, understand the experiences of people with HIV related to the COVID-19 pandemic, and improve our understanding of the experiences of gender diverse individuals regarding HIV care and prevention. The Florida Cohort study also offered opportunities for ancillary data collection for other projects and will provide access to deidentified data for additional analyses.

The sample enrolled in wave 3 was similar to the overall population of people with HIV living in Florida [5]. There were a few exceptions to this. First, cisgender women and transgender people were overrepresented in the Florida Cohort study sample compared to their distribution statewide. Second, Hispanic people with HIV were underrepresented in wave 3. Although almost all questionnaires were available in Spanish, enrollment and data collection in Spanish were limited to 2 recruitment sites (Polk and Miami-Dade counties) that had a Spanish-speaking research assistant. Third, enrollment was higher in north and central Florida, which have lower overall prevalence of Hispanic people, than in south Florida, which has higher rates and total numbers of people with HIV [52]. Finally, the Florida Cohort study did not enroll people with HIV aged below 18 years. We did not enroll people with dementia or living in nursing homes. Therefore, adolescents and persons aged 70 or older were underrepresented.

The Florida Cohort study wave 3 includes participants recruited from a network of clinics and organizations across the state. Unlike the previous wave, which focused largely on recruitment from FDOH clinics, wave 3 included a wider range of community clinics, case management agencies, and recruitment from the public. The Florida Cohort study wave 3 also included several counties that are listed as priority jurisdictions in the EHE initiative [3]. This wave also made a concerted effort to recruit key populations and surpassed the previous wave in recruitment of those who met the criteria for at-risk drinking, individuals who identified as transgender or gender nonconforming, and participants from rural areas, although the target of 150 was not met. Furthermore, we successfully enrolled a subset of participants who spoke Haitian Creole who had not been included in previous waves of the Florida Cohort study but will be helpful in understanding and addressing HIV disparities. People with HIV of Haitian descent are more likely to be diagnosed at a later stage and not achieve key steps in the HIV care continuum [53-55].

A major challenge to enrollment, especially during the first year of the study, was the COVID-19 pandemic and changing mitigation strategies implemented across the state at different times. This study was originally slated to start in March 2020, but the pandemic delayed the initiation of the study until October 2020. This study relied heavily on recruitment through clinics, which shifted to telehealth during the pandemic. Moreover, the management and staff at clinics that maintained in-person visits were understandably hesitant to allow recruiters into clinical spaces. In response, the team shifted to remote recruitment, which was generally less successful than in-person recruitment. Relying on remote recruitment methods where clinical staff served as intermediaries led to lower-than-expected enrollment in the first year of the study for all active sites, and this pattern continued in Miami-Dade County, where the COVID-19 restrictions lasted the longest.

Many lessons were learned during the implementation of this study. To reach a wider range of people with HIV and help the study better integrate into the clinic flow in a variety of settings, this study offered participants many ways to enroll, complete the surveys, and receive compensation. Although this worked well for many participants and sites, it also greatly complicated research staff training and the administration of the study. In addition, some of these methodologies worked better than others. For example, sites that offered consent forms, questionnaires, and payments via US mail frequently encountered problems with items being lost or participants returning either the consent forms or the questionnaires but not both. Once clinics began seeing patients in person again and allowed recruiters back into the clinic, the study team found that obtaining consent from participants in person and following up with a link to the questionnaires worked the best for most participants.

Follow-up rates for the 12-month questionnaire were lower than desired, although follow-up questionnaire data were not needed to answer the key study research questions. To improve follow-up rates, we would benefit from more consistent staffing and strategies to try to keep people more engaged in the study. We had limited face-to-face connections with people, and that could have also impacted follow-up rates. We successfully integrated new questionnaire items and collected information that individual researchers and graduate students could use for their research. At least 3 faculty members are using the Florida Cohort study data to support their research funded by National Institutes of Health individual career development awards, and at least 5 graduate students in epidemiology and nursing are using the data as part of their doctoral dissertations. This study offered opportunities for the research team to engage with clinic staff and HIV service team members across the state. By working with multiple clinic settings and locations, the investigators could learn about issues that were emerging in the community and that could be added to our questionnaires. Community members also helped us update our stigma questionnaire.

In the future, coded data without direct identifiers from this study may be linked to other datasets using a hash ID number, created by a formula that uses direct identifiers but cannot readily identify an individual. Subsequently, all ID numbers, dates, and codes can be removed to prepare a completely deidentified dataset. With IRB review and approval, this procedure has been tested linking data from the Florida Cohort study questionnaires to clinical, dispensing and claims data from the OneFlorida+ Data Trust, which includes medical information for more than 20 million patients on both public and private insurance [56], and the Substance Abuse Mental Health Information System, which includes information on substance use and mental health treatment.

### Limitations

Our team used different recruitment strategies across different recruitment sites; therefore, it is not appropriate to compare any health behaviors or outcomes specifically by recruitment setting. Because we used a convenience sampling method, and non-English questionnaires were not available at all sites, any comparisons across race and ethnicity will need to be done with caution. In addition, several items, including ART preferences and COVID-19 vaccinations, were updated over time, limiting the ability to assess these constructs longitudinally. However, key measures such as symptoms of depression and anxiety and alcohol use remained the same.

### Conclusions

In summary, the Florida Cohort study wave 3 enrolled a diverse group of people with HIV from several sites around the state, including both EHE and non-EHE counties. The data can be used to help address key barriers to steps in the HIV care continuum and achieve the goals of the EHE initiative within the state. The Florida Cohort study wave 4 began enrollment in 2024 and will include many of the core measures from wave 3 with additional items collecting formative data to inform alcohol and mental health interventions and enrolling people with HIV from new recruitment locations.

## Appendix

Multimedia Appendix 1 CHERRIES checklist.

Multimedia Appendix 2 STROBE checklist.

Multimedia Appendix 3 Peer review report from ZAA1 DD (10) - National Institute on Alcohol Abuse and Alcoholism Special Emphasis Panel CHAART Consortium RFA (National Institutes of Health, USA).

## Abbreviations

ART: antiretroviral therapy
CHERRIES: Checklist for Reporting Results of Internet E-Surveys
eHARS: Enhanced HIV/AIDS Reporting System
EHE: Ending the HIV Epidemic
FDOH: Florida Department of Health
IRB: institutional review board
REDCap: Research Electronic Data Capture
SHARC: Southern HIV and Alcohol Research Consortium
STROBE: Strengthening the Reporting of Observational Studies in Epidemiology
UF: University of Florida

## Data Availability

The questionnaire datasets generated and analyzed during this study are available from the Southern HIV and Alcohol Research Consortium Concepts System on reasonable request. Requests should be submitted via the Southern HIV and Alcohol Research Consortium Concepts System [57]. Data from the Enhanced HIV/AIDS Reporting System is not publicly available due to Florida Department of Health policy but can be requested from the Florida Department of Health. Access to the Enhanced HIV/AIDS Reporting System data requires a separate data use agreement.
