# Supplementary material for: Identifying Factors Associated With HIV Viral Suppression and Health Care Outcomes in the Florida Cohort Study Wave 3: Protocol for a Prospective Cohort Study
Source: JMIR Res Protoc. 2025 Sep 25;14:e69702. doi: 10.2196/69702 (PMC12511819; doi:10.2196/69702)
Supplement: Multimedia Appendix 3 [file resprot_v14i1e69702_app3.pdf]

SUMMARY STATEMENT
(Privileged Communication)

PROGRAM CONTACT:
Dr. Deidra Roach
301-443-5820
droach@mail.nih.gov

Release Date: 05/09/2016
Revised Date:

Application Number: 2 U24 AA022002-06

Principal Investigator

COOK, ROBERT L

Applicant Organization: UNIVERSITY OF FLORIDA

Review Group:	ZAA1 DD (10)<br>National Institute on Alcohol Abuse and Alcoholism Special Emphasis Panel<br>CHAART Consortium RFA<br>AIDS - EXP. REV.
Meeting Date:	04/27/2016
Council:	MAY 2016
Requested Start:	07/01/2016
RFA/PA:	AA16-002
PCC:	ACAD

Project Title:	Southern HIV and Alcohol Research Consortium Administrative and Research Support Core
SRG Action:	Impact Score:24
Next Steps:	Visit http://grants.nih.gov/grants/next_steps.htm
Human Subjects:	30-Human subjects involved - Certified, no SRG concerns
Animal Subjects:	10-No live vertebrate animals involved for competing appl.
Gender:	1A-Both genders, scientifically acceptable
Minority:	1A-Minorities and non-minorities, scientifically acceptable
Children:	1A-Both Children and Adults, scientifically acceptable<br>Clinical Research - not NIH-defined Phase III Trial

Project Year	Direct Costs Requested	Estimated Total Cost
6	401,717	598,178
7	421,616	627,809
8	427,433	636,471
9	433,425	645,393
10	439,600	654,588
TOTAL	2,123,791	3,162,438

ADMINISTRATIVE BUDGET NOTE: The budget shown is the requested budget and has not been adjusted to reflect any recommendations made by reviewers. If an award is planned, the costs will be calculated by Institute grants management staff based on the recommendations outlined below in the COMMITTEE BUDGET RECOMMENDATIONS section.

2U24AA022002-06 Cook, Robert

RESUME AND SUMMARY OF DISCUSSION: This is a renewal U24 application submitted in response to RFA-AA-16-002 titled “Limited Competition: Consortia for HIV/AIDS and Alcohol–Related Research Trials (CHAART) Administrative Resource Core” and supports the Southern HIV Alcohol Research Consortium (SHARC). This Administrative and Research Infrastructure Core proposes to provide continued leadership, collaboration, and scientific oversight for all activities related to SHARC. The progress in previous funding has been impressive and has recruited over 600 subjects for the Florida Cohort Study, and has helped generate several grants and papers, in addition to serving as a valuable resource for new research scientists. The organizational structure is sound, and the development of a data management system has enhanced the functionality. The collaboration with the proposed Resource core is new for this funding period. Likewise, the research objectives of the companion U01 research projects are also new, and there is limited description of how the admin core integrates and supports the companion resource core and U01 research studies. However, these weaknesses were regarded as minor, and the application was rated in the excellent to outstanding range.

DESCRIPTION The Southern HIV Alcohol Research Consortium (SHARC) was established in 2012 as one of five national Consortia for HIV/AIDS and Alcohol Research Translation. The mission of SHARC is to improve health outcomes and reduce HIV transmission in persons affected by alcohol and HIV in Florida. Florida has the highest rate of new HIV infections in the US, a growing proportion of HIV+ persons over age 50, and broad population diversity across age, gender, race/ethnicity, and geography (rural/urban). The next phase of SHARC (2016-2021) will focus on the relationship of alcohol to HIV transmission (including HIV viral suppression) and HIV comorbidities (especially brain function and liver disease). SHARC will consist of three integrated proposals that will: centralize our administrative and research infrastructure (this U24), determine whether harmful aspects of alcohol on the brain are reversible (U01), and provide expertise in strategies to change drinking behavior and in biostatistical analyses (U24). Specifically, this U24 proposal will centralize SHARC administrative services, and also continue represents the Administrative the Florida Cohort study that backbone of SHARC infrastructure and collaborative activity. The Cohort will be the source of participants for our U01 research, and both the Cohort and U01 projects will be supported by the U24 behavioral/biostatistical core. The specific aims of this U24 are Aim 1: Ensure leadership, collaboration, and scientific oversight for all activities related to SHARC. Specifically, the Core will: obtain input from an Executive Committee, a Scientific Advisory Board, and a Community Advisory Board; monitor our scientific contributions and research productivity, manage day-to-day operations, coordinate and facilitate requests to access SHARC data and/or biological samples, provide IRB and regulatory support, and communicate our scientific findings. Aim 2: Support ongoing collaborative research activity related to the Florida Cohort. The Cohort was initiated in 2014 and will complete enrollment of 1700 persons (1500 HIV+, 200 HIV-). Of these, 900 HIV+ will be tracked prospectively using enhanced measures of alcohol consumption, neurocognition, liver disease, and HIV viral suppression. These 900 will include 300 with heavy drinking (> 7 drinks/week women, >14 drinks/week men), 300 with moderate drinking, and 300 with no current drinking. The Florida Cohort represents the backbone of SHARC infrastructure that allows us to a) engage in collaborative research that links public health settings and academic institutions; b) support and expand our Training and Development Program; and c) facilitate recruitment of participants into our companion U01 via a participant contact registry. The wide range of drinking behavior and population diversity within the Cohort will allow us to compare the impact of heavy drinking on HIV outcomes across diverse populations and communities. By partnering with at least 8 distinct public health clinics and settings across the state, we are now poised to collaborate on a range of intervention and implementation studies targeting individuals and public health clinics and settings.

PUBLIC HEALTH RELEVANCE
The mission of the Southern HIV Alcohol Research Consortium (SHARC) is to improve health outcomes and reduce HIV transmission among the diverse populations affected by alcohol and HIV in

the Southeastern US. This Administrative Core will have direct public health relevance by providing the structured infrastructure, access to research participants and data, and research training needed to address this mission.

CRITIQUE 1:

Significance: 3
Investigator(s): 4
Innovation: 3
Approach: 4
Environment: 1

Overall Impact:

The focus of the Southern HIV Alcohol Research Consortium (SHARC) is to improve health outcomes and reduce HIV transmission in persons affected by alcohol and HIV in Florida. The next phase of SHARC will emphasize the relationship of alcohol to HIV transmission/viral suppression and HIV comorbidities (especially brain function and liver diseases). This proposal is for the Administrative and Research Infrastructure Core. Aim 1 is to ensure leadership, collaboration, and scientific oversight for all activities related to SHARC. Specifically, the Core will: obtain input from an Executive Committee, a Scientific Advisory Board, and a Community Advisory Board; monitor scientific contributions and research productivity, manage day-to-day operations, coordinate and facilitate requests to access SHARC data and/or biological samples, provide IRB and regulatory support, and communicate scientific findings. Aim 2 is to support ongoing collaborative research activity related to the Florida Cohort.

Overall, this is a strong application to provide administrative support to SHARC. They have demonstrated success in the previous funding period with regard to publications and grant submissions, as well as creating mentorship opportunities. The Florida Cohort is a success, and there are plans to add to the cohort (which is necessary to increase the number of Hispanics, MSM and persons over 60). The organizational structure is sound, and the development of a data management system has enhanced the functionality. The investigative team is strong, but could benefit from more behavioral science expertise, and expertise with gender and sexual minority populations. Plans for the "Enhanced Cohort" need clarification – as specific research questions related to the need for this cohort are vague. SHARC has been successful in working with academic/research institutions, departments of health, and community-based agencies. There are some minor weaknesses, but these do not dampen enthusiasm.

1. Significance:

Strengths

- Rates of HIV infection in Florida are high, and the state represents an important area in which to conduct HIV-alcohol relevant research.

Weaknesses

- Throughout the application, there is insufficient consideration of the unique HIV-alcohol issues facing gender and sexual minorities, particularly MSM and transgender women who are disproportionally affected by HIV.

2. Investigator(s):

Strengths

- The PI has the relevant expertise and experience to lead this Core. His contributions to our understanding of alcohol and HIV infection have been significant.

Weaknesses

- The contributions of Co-Investigators are not clearly articulated.
- The team has limited behavioral science expertise, aside from one Co-Investigator (Whithead) who is quite junior with limited publications.
- The team would benefit from expertise in gender and sexual minorities in order to ensure that sufficient numbers are enrolled in the Florida Cohort to reflect the rates of these populations in the state of Florida.

3. Innovation:

Strengths

- Some elements of the data management and information system are innovative.
- The Florida Cohort is innovative and includes the under-researched Haitian population.

Weaknesses

- The idea of an Enhanced Cohort of 900 HIV+ persons (focused on alcohol issues) is a good one, but specific hypotheses and plans for this cohort are not well articulated.

4. Approach:

Strengths

- A Data management and information system to support data sharing and analyses was developed and is now available to support SHARC.
- During the first phase of funding, SHARC was successful in terms of grant/paper submissions and funding. The Professional Development Program should sustain this success moving forward.
- Faculty involved are diverse, and enable preparation of materials in Spanish and Haitian Creole.
- The Organizational Structure is strong – guided by an Executive Committee of 7 members, a Scientific Advisory Board, and a Community Advisory Board.
- The seminar series and annual conference appear to have generated positive response and have helped to promote mentorship/training.

Weaknesses

- Preliminary data from the Florida Cohort is impressive, yet no specific findings regarding MSM – who are disproportionally affected by HIV – are presented.
- Although it is recognized that there are too few MSM enrolled in the cohort, specific plans for how MSM will be targeted for inclusion moving forward are not included. It is likely this is due to the lack of expertise in gender and sexual minorities on the investigative team.

5. Environment:

Strengths

- The facilities are excellent, and well detailed. Institutional support for the SHARC renewal is strong and includes funding support and research space.

- Recruitment settings are also clearly delineated and include academic/research partnerships, health departments, and community-based centers.

Weaknesses

- None noted.

Protections for Human Subjects:

Acceptable Risks and/or Adequate Protections

Data and Safety Monitoring Plan (Applicable for Clinical Trials Only):

Acceptable

Inclusion of Women, Minorities and Children Applicable Only for Human Subjects Research:

G1A - Both Genders, Acceptable

M1A - Minority and Non-minority, Acceptable

C3A - No Children Included, Acceptable

Budget and Period of Support:

- Dr. Cook's base salary, and that of Dr. Nelson, exceed the NIH cap.

CRITIQUE 2:

Significance: 1
Investigator(s): 1
Innovation: 2
Approach: 2
Environment: 1

Overall Impact:

This U24 administrative proposal supports the Southern HIV Alcohol Research Consortium (SHARC) that was established in 2011 by integrating its administrative services. The proposal aims at providing oversight for the operational aspects of SHARC and facilitate the examination of the role of alcohol abuse in HIV pathogenesis, particularly, the development of HIV co-morbidities involving brain function and liver disease. To assist and coordinate the scientific endeavor of SHARC, this administrative U24 proposal will continue to support and sustain the Florida Cohort study which is the mainstay of the SHARC research infrastructure and collaborative activity. Overall, the core has the capabilities of providing effective administrative management to support and sustain the proposed studies.

1. Significance:

Strengths

- Administratively manages and continues to support and build upon the observational and intervention research infrastructure initiated in the first round of CHAART.
- Supports SHARC mission to improve health outcomes and reduce HIV transmission among the diverse population affected by alcohol abuse and HIV infection in the Southeastern United States, by coordinating research, training, and collaborative activities.

- Play a significant role in facilitating the implementation of alcohol interventions into a range of HIV clinical and public health settings across Florida.

Weaknesses

- None noted

2. Investigator(s):

Strengths

- The PI – Dr. Cook is a physician scientist with a significant experience in the proposed area of research and is highly qualified to serve as the director and steer the operational and scientific activities involved in the SHARC administrative and research support core.
- The PI is amply supported by an excellent team of co-investigators/collaborators and is certainly a major strength.

Weaknesses

- None noted.

3. Innovation:

Strengths

- Establishment and development of the Florida Cohort that represents diverse HIV populations across the state of Florida and US is clinically significant and innovative.
- Program for the development of junior investigators through multidisciplinary experiences, hands-on research activity, and community engagement is also relevant and innovative.

Weaknesses

- None noted.

4. Approach:

Strengths

- Expansion of the Florida Cohort from the current 600 persons with HIV to 1700 persons, of whom 900 will be recruited and tracked prospectively using enhanced measures and annual follow-ups related to alcohol, neurocognition, and liver disease.
- The newly recruited cohort of 900 HIV persons with HIV to be comprised of individuals representing a spectrum of alcohol abuse pattern from heavy, moderate to no current drinking. This will allow comparison of drinking outcomes according to drinking status.
- Formation of a new data management and information system to support data sharing and analyses.
- Establishment of standardized policies and procedures for internal review and approval, authorship guidelines, and formal data sharing agreements.
- Facilitating the access and availability of Florida Cohort data, participants, and bio-specimens supporting research, training and collaborative activity.

Weaknesses

- None noted.

5. Environment:

Strengths

- Excellent resources and adequate infrastructure that can support large clinical trials and implementation of proposed alcohol intervention projects.

Weaknesses

- None noted

Protections for Human Subjects:

Acceptable Risks and/or Adequate Protections

Data and Safety Monitoring Plan (Applicable for Clinical Trials Only):

Acceptable

Inclusion of Women, Minorities and Children Applicable Only for Human Subjects Research:

G1A - Both Genders, Acceptable

M1A - Minority and Non-minority, Acceptable

C3A - No Children Included, Acceptable

Vertebrate Animals:

Not Applicable (No Vertebrate Animals)

Biohazards:

Acceptable

Renewal:

- The U24 Administrative and Research Support Core is part of a competitive renewal of the existing Southern HIV Alcohol Research Consortium (SHARC).

Resource Sharing Plans:

- Acceptable

Budget and Period of Support:

- Recommend as Requested

CRITIQUE 3:

Significance: 1
Investigator(s): 1
Innovation: 3
Approach: 2
Environment: 1

Overall Impact:

The Southern HIV Alcohol Research Consortium (SHARC) will provide administrative and research infrastructure for the companion Resource core and research project. The main accomplishment of the core is the creation of the Florida Cohort Study, which will enroll 1700 individuals, of whom ~600 were recruited during the initial funding period. In addition to the Florida Cohort Study, there was impressive progress for SHARC during the initial funding period with many credited grants and papers, and serving as a valuable resource for new research scientists. One concern was the limited description regarding specifically how the admin core will serve the companion research projects (U01). The collaboration with the Resource core also appears to be new for this funding period, thus additional description of how that collaboration will work is needed.

1. Significance:

Strengths

- The Florida Cohort Study has enrolled high-risk patients and provides an important resource for key research in the HIV/alcohol field.

Weaknesses

- None noted.

2. Investigator(s):

Strengths

- Highly qualified and well-established PI, strong co-investigators, with support from a Scientific Advisory Committee and Community Advisory Committee.

Weaknesses

- None noted.

3. Innovation:

Strengths

- Although the administrative core is not innovative by itself, it provides an important resource for innovative research, such as the evaluation of alcohol use on brain and cognitive function

Weaknesses

- None noted.

4. Approach:

Strengths

- Well-designed cohort study serving as key resource for ongoing research.
- Well integrated within the University of Florida providing opportunity for collaborations, including new scientists. Annual conference sponsored by SHARC is an excellent approach to disseminate key findings and foster collaborative environment.

Weaknesses

- Limited description how the core integrates and supports the companion resource core and U01 research studies.

5. Environment:

Strengths

- Excellent environment at University of Florida to support all administrative and research activities of SHARC.

Weaknesses

- None noted.

Protections for Human Subjects:

- Acceptable Risks and/or Adequate Protections

Data and Safety Monitoring Plan (Applicable for Clinical Trials Only):

Not Applicable (No Clinical Trials)

Inclusion of Women, Minorities and Children:

- Sex/Gender: Distribution justified scientifically
- Race/Ethnicity: Distribution justified scientifically
- Inclusion/Exclusion of Children under 21: Including ages < 21 justified scientifically

Resource Sharing Plans:

Acceptable

Budget and Period of Support:

- Recommend as Requested

THE FOLLOWING SECTIONS WERE PREPARED BY THE SCIENTIFIC REVIEW OFFICER TO SUMMARIZE THE OUTCOME OF DISCUSSIONS OF THE REVIEW COMMITTEE, OR REVIEWERS' WRITTEN CRITIQUES, ON THE FOLLOWING ISSUES:

PROTECTION OF HUMAN SUBJECTS (Resume): ACCEPTABLE

INCLUSION OF WOMEN PLAN (Resume): ACCEPTABLE

INCLUSION OF MINORITIES PLAN (Resume): ACCEPTABLE

INCLUSION OF CHILDREN PLAN (Resume): ACCEPTABLE

COMMITTEE BUDGET RECOMMENDATIONS: The budget was recommended as requested.

Footnotes for 2 U24 AA022002-06; PI Name: Cook, Robert L

NIH has modified its policy regarding the receipt of resubmissions (amended applications). See Guide Notice NOT-OD-14-074 at http://grants.nih.gov/grants/guide/notice-files/NOT-OD-14-074.html. The impact/priority score is calculated after discussion of an application by averaging the overall scores (1-9) given by all voting reviewers on the committee and multiplying by 10. The criterion scores are submitted prior to the meeting by the individual reviewers assigned to an application, and are not discussed specifically at the review meeting or calculated into the overall impact score. Some applications also receive a percentile ranking. For details on the review process, see http://grants.nih.gov/grants/peer_review_process.htm#scoring.

MEETING ROSTER
National Institute on Alcohol Abuse and Alcoholism Special Emphasis Panel

NATIONAL INSTITUTE ON ALCOHOL ABUSE AND ALCOHOLISM
CHAART Consortium RFA
ZAA1 DD (10)
04/27/2016 - 04/29/2016

CHAIRPERSON(S)

PARSONS, JEFFREY T, PHD
DISTINGUISHED PROFESSOR
DIRECTOR, CENTER FOR HIV/AIDS EDUCATIONAL STUDIES AND TRAINING (CHEST)
HUNTER COLLEGE AND THE GRADUATE CENTER
OF THE CITY UNIVERSITY OF NEW YORK
NEW YORK, NY 10065

MEMBERS

BAGBY, GREGORY JOHN, PHD
PROFESSOR
DEPARTMENT OF PHYSIOLOGY
LSU HEALTH SCIENCES CENTER
NEW ORLEANS, LA 70112-1393

BALACHOVA, TATIANA N, PHD
ASSOCIATE PROFESSOR
DEPARTMENT OF PEDIATRICS
CHILD STUDY CENTER
THE UNIVERSITY OF OKLAHOMA HEALTH SCIENCES CENTER
OKLAHOMA CITY, OK 73117

BALASUBRAMANIAN, RAJI, DSC
ASSOCIATE PROFESSOR
DIVISION OF BIOSTATISTICS AND EPIDEMIOLOGY
SCHOOL OF PUBLIC HEALTH AND HEALTH SCIENCES
UNIVERSITY OF MASSACHUSETTS
AMHERST, MA 01003

BARVE, SHIRISH S, PHD
PROFESSOR
DEPARTMENT OF MEDICINE
AND PHARMACOLOGY AND TOXICOLOGY
UNIVERSITY OF LOUISVILLE
LOUISVILLE, KY 40202

CLARK, URAINA S, PHD
DEPARTMENT OF NEUROLOGY
ICAHN SCHOOL OF MEDICINE AT MOUNT SINAI
NEW YORK, NY 10029

DAVIS, KELLY CUE, PHD
RESEARCH ASSOCIATE PROFESSOR
SCHOOL OF SOCIAL WORK
UNIVERSITY OF WASHINGTON
SEATTLE, WA 98105

DOWDY, DAVID WESLEY, MD, PHD
ASSISTANT PROFESSOR
DEPARTMENT OF EPIDEMIOLOGY
BLOOMBERG SCHOOL OF PUBLIC HEALTH
JOHNS HOPKINS UNIVERSITY
BALTIMORE, MD 21205

KALICHMAN, SETH C, PHD
PROFESSOR
DEPARTMENT OF PSYCHOLOGY
UNIVERSITY OF CONNECTICUT
STORRS, CT 06269

KERR, WILLIAM C, PHD
SENIOR SCIENTIST
ALCOHOL RESEARCH GROUP
PUBLIC HEALTH INSTITUTE
EMERYVILLE, CA 94608

KRAEMER, KEVIN L, MD
ASSOCIATE PROFESSOR
HEALTH POLICY AND MANAGEMENT
SCHOOL OF PUBLIC HEALTH
UNIVERSITY OF PITTSBURGH
PITTSBURGH, PA 15213

MCPHERSON, STERLING M., PHD
ASSISTANT PROFESSOR
COLLEGE OF NURSING PROGRAM OF
EXCELLENCE IN ADDICTIONS RESEARCH
WASHINGTON STATE UNIVERSITY
SPOKANE, WA 99210

O'DONNELL, MAX, MD
ASSISTANT PROFESSOR OF MEDICINE
DEPARTMENT OF MEDICINE AND EPIDEMIOLOGY
COLUMBIA UNIVERSITY
NEW YORK, NY 10461

PALFAI, TIBOR P., PHD
ASSOCIATE PROFESSOR
DEPARTMENT OF PSYCHOLOGY
BOSTON UNIVERSITY
BOSTON, MA 02215

SILVERBERG, MICHAEL J, PHD
RESEARCH SCIENTIST
DIVISION OF RESEARCH
KAISER PERMANENTE
OAKLAND, CA 94612

ZAHR, NATALIE M, PHD
RESEARCH SCIENTIST
DEPARTMENT OF HEALTH SCIENCES
SRI INTERNATIONAL
MENLO PARK, CA 94025

SCIENTIFIC REVIEW OFFICER

SRINIVAS, RANGA, PHD
CHIEF, EXTRAMURAL PROJECT REVIEW BRANCH
EXTRAMURAL PROJECT REVIEW BRANCH
NATIONAL INSTITUTE ON ALCOHOL ABUSE AND ALCOHOLISM
NATIONAL INSTITUTES OF HEALTH
ROCKVILLE, MD 20855

EXTRAMURAL SUPPORT ASSISTANT

FULTON, THELMA
EXTRAMURAL SUPPORT ASSISTANT
OFFICE OF EXTRAMURAL ACTIVITIES
NATIONAL INSTITUTE ON ALCOHOL ABUSE AND ALCOHOLISM
NATIONAL INSTITUTES OF HEALTH
ROCKVILLE, MD 20892-9304

Consultants are required to absent themselves from the room during the review of any application if their presence would constitute or appear to constitute a conflict of interest.
